# A new approach for the pre-clinical optimization of a spatial configuration of bifurcated endovascular prosthesis placed in abdominal aortic aneurysms

**DOI:** 10.1371/journal.pone.0182717

**Published:** 2017-08-09

**Authors:** Andrzej Polanczyk, Aleksandra Piechota-Polanczyk, Ludomir Stefańczyk

**Affiliations:** 1 Department of Heat and Mass Transfer, Faculty of Process and Environmental Engineering, Lodz University of Technology, Lodz, Poland; 2 Department of Medical Biotechnology, Faculty of Biochemistry, Biophysics and Biotechnology, Jagiellonian University, Krakow, Poland; 3 Department of Radiology and Diagnostic Imaging, Medical University of Lodz, Lodz, Poland; Medical University Innsbruck, AUSTRIA

## Abstract

Complexity of the spatial configuration of an aortic implant with bifurcation in the distal part is related to changes in blood hemodynamic in the area of bifurcation which may disturb blood flow and lead to thrombus formation. This study was designed to characterize parameters which define spatial configuration of an aortic implant for which the risk of thrombus formation is the smallest. We used AngioCT data from 74 patients, aged 55 ±10 years, after endovascular procedure to prepare 3D geometries of stent-grafts. Computational Fluid Dynamics (CFD) simulations were used to reconstruct blood hemodynamic and simulate thrombus formation. Next, geometric parameters of stent-grafts included the ratio of volume of upper part to the bifurcations, the relation of inlet and outlet diameters of a stent-graft and deformations in the iliac part of the stent-graft were analyzed. We also analyzed tortuosities (spiral twisting of the flow around the flow direction) and bends (the largest angulation in distal part of a stent-graft). The CFD results were confronted with AngioCT data to verify if computer generated thrombus appeared in particular patient. Additionally, geometric parameters of analyzed stent-grafts were used to propose a mathematical tool for prediction of thrombus appearance. The results showed that tortuosities and bends of a stent-graft had the highest impact on thrombus formation. Formation of thrombi was observed in 22% to 31% of cases (at blood hematocrit *Hct* = 40%) even for small values of tortuosities and bends indicating that these parameters are dominant in determining blood clotting. Our calculated results overlapped with clinical data in 80% to 91%. Therefore, we conclude that tortuosities and bends have high impact on thrombus formation and should be under special attention during stent-graft recommendation and patients’ follow-ups.

## Introduction

One of the commonly applied methods for reconstruction of proper blood flow in vessel is implantation of endovascular prosthesis (stent-graft) in the area of wall dissection or abdominal aortic aneurysm (AAA) [1, 2].

The implantation of stent-graft decreases the pressure acting directly on the vessel’s wall by accumulating energy carried by pulsation wave. This effect may reduce the probability of wall rapture in the area of aneurysm appearance. [3–6]. Although stent-graft implementation has numerous benefits it may have some negative clinical outcome over time. The possible post-operative complications include stent-graft endoleak, conformational and/or morphological changes in the endograft precipitating kinking or even thrombus development. All of those complications were previously described by others [7, 8]. When not diagnosed on time thrombus may lead to and/or limb occlusion of blood flow through implant’s leg leading to ischemia of the lower limb and eventually even to amputation [9–11]. Therefore, early diagnosis of thrombus formation or post-operative assessment of thrombus-prone areas in stent-graft with computer simulations may be useful in early diagnosis [12, 13].

Moreover, complexity of the construction of endovascular prosthesis for AAA area, connected with the bifurcation in the distal part (branching of iliac arteries), may lead to blood flow disturbances and further to thrombus development [14]. Therefore, before implantation of endovascular prosthesis AAA size and spatial configuration have to be assessed to select a proper type of a stent-graft [2]. Initial diagnosis require the assessment of few diameters including the diameter of the aorta below, above and at the level of the renal arteries, the diameter of aneurysm sac, and the diameter of common and external iliac arteries. Often there is a need to assess the size of thrombus in the aneurismal sac [15]. Hence, the stent-graft size has to be choose carefully, as studies reported that an improper selection of aortic implant may lead to blood stagnation and eventually to thrombus formation which further hampers blood flow through the stent-graft duct [7, 13].

A selection of an optimum shape of the endovascular prosthesis is undertaken during surgery planning. Therefore, optimization of the stent-graft's spatial configuration seems to be an important step. In the human body, the angles and diameters of bifurcating vessels are optimized in a natural way to improve blood flow hemodynamic. The angle at which blood vessels are bifurcating is approximately 45° and the ratio of diameters of branched blood vessels is around 0.8 [16].

Many authors analyze the mechanical aspects of the endovascular prosthesis with the Computational Fluid Dynamic (CFD) technique [17–19]. Currently, in many works the CFD technique is applied to assess blood flow hemodynamic in vessels after stent-graft implantation in an aneurysm [20].

Therefore, the aim of this study was to determine which part of the stent-graft has the highest impact on the thrombus formation and to propose a pre-operative method of optimization of the spatial configuration of endovascular prosthesis.

## Material and methods

### 3D reconstructions of endovascular prosthesis

In this study we used data collected form 74 patients aged 55 ±10 years after AngioCT (GE Light-Speed 64 VCT; GE Healthcare, Fairfield, CT, USA) who underwent treatment with endovascular procedure in the Barlicki Hospital No. 2 in Lodz (Poland) from 2007 to 2016 (Table 1). Patients were implanted with one of the three different types of stent-grafts: Zenith made by COOK (Cook Medical, USA), Endurant made by Medtronic (Medtronic, USA) and Excluder made by Gore (Gore, USA). The type of stent-graft affects the spatial configuration of the flow area, which is rendered as a space filled with blood with the contrast agent. The mechanical properties of the wall are similar regardless the type of an implant [21]. The scaffold of Zenith and Endurant stent-grafts is made of openwork metal segments which do not overlap. The cover is made of Dacron (Polyester material). The scaffold of Excluder stent-graft is built with metal elements that partly overlap in order to prevent angular corners. The coating is made of PTFE (Polytetrafluoroethylene). The Zenith segments are slightly wider (20mm) whereas the Endurant parts are narrower (from 15 to 18mm). This feature is independent of the length of the stent-graft and its diameter. Moreover it may have an effect on the better fit in the tortuous vessels due to potentially greater flexibility. The profile of the delivery system, depended on the construction of the stent-graft, but our research was retrospective after implantation and concerned the stability of the achieved effect [10].

**Table 1:** Clinical data description.

Total number of patients	Patients without thrombus	Patients with thrombus
74	23	51

Patients’ data were retrospectively collected after obtaining written informed consent to participate in the study. Medical data and images were anonymized by coding information before access and analysis. The study was approved by the Local Ethic Committee on Medical University of Lodz (RNN/126/07/KE).

Using a specialized 3DDoctor software (Able Software Corp., Lexington, MA, USA) for medical image processing, three-dimensional reconstructions of the analyzed stent-grafts were performed.

The 3d geometries were prepared so that in each case the inlet to the system was just below the mesenteric artery outlet and the outlets were located in the femoral arteries at the level below the stent-graft ending. Numerical calculations of blood flow in the analyzed geometries were carried out with the use of ANSYS FLUENT.12.1 software (ANSYS, USA). The mathematical domain was described with the boundary conditions as follow: (1) the stent-graft wall was treated as a rigid structure, (2) at the inlet, an assumed condition of blood flow velocity was u = (u,v,w), (3) while at the outlets the condition p = const was taken. Blood was treated as a non-Newtonian described in details in our previous paper [22]. For the purpose of optimization of stent-graft spatial configuration we used for each case blood hematocrit (*Hct*) ranging from 30% to 60%. The process of thrombus appearance/formation was calculated with the use of modified Quemada's model, as previously described in Polanczyk et al. [22]. The blood flow velocity profiles for each case were obtained from the USG-Doppler tests (GE Vivid 7, GE Healthcare, USA) performed in the Barlicki Hospital No. 2 in Lodz (Poland). Velocity profiles determined from USG-Doppler data were further used as a boundary condition at the inlet to the particular calculation domain. All CFD results were confronted with clinical data.

### Verification method

Medical data base created for the purpose of this study consisted of patients with and without thrombus diagnosed in endovascular prosthesis. Number of patients with diagnosed thrombus was 51. This allowed verification of proposed methodology of optimization of spatial configuration of endovascular prosthesis. Analyzed patients had *Hct* at level 40%, and for this value a verification process was made. Higher value of *Hct* was applied as a prognostic tool to check how particular spatial configuration of a stent-graft behaves.

### Optimization of spatial configuration of stent-grafts

**Fig 1:**
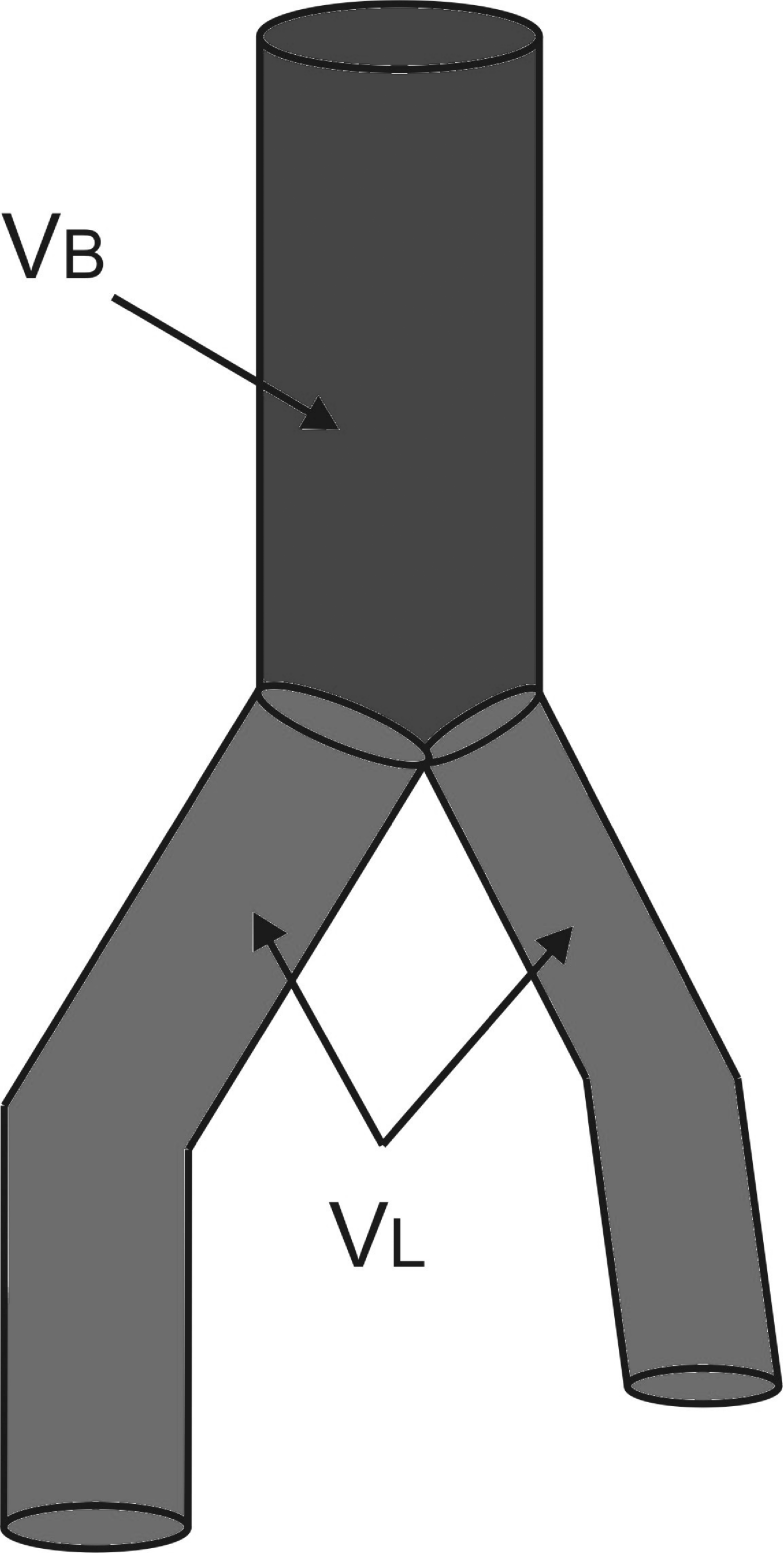
Schematic representation of volumes analyzed in the stent-graft. *V*_*B*_−volume of the upper part of the stent-graft marked in blue, *V*_*L*_−volume of the iliac part of the stent-graft marked in green.

To optimize the spatial configuration of the endovascular prosthesis, the following geometric parameters were analyzed: the stent-graft volume, inlet and outlet diameters of the stent-graft and, deformations of the iliac part of stent-graft. Fig 1 shows schematically the analyzed volumes of the stent-graft described as a ratio of upper part (common part) (*V*_*B*_*)* to the volume of the iliac part of the endovascular prosthesis (*V*_*L*_*)*.

**Fig 2:**
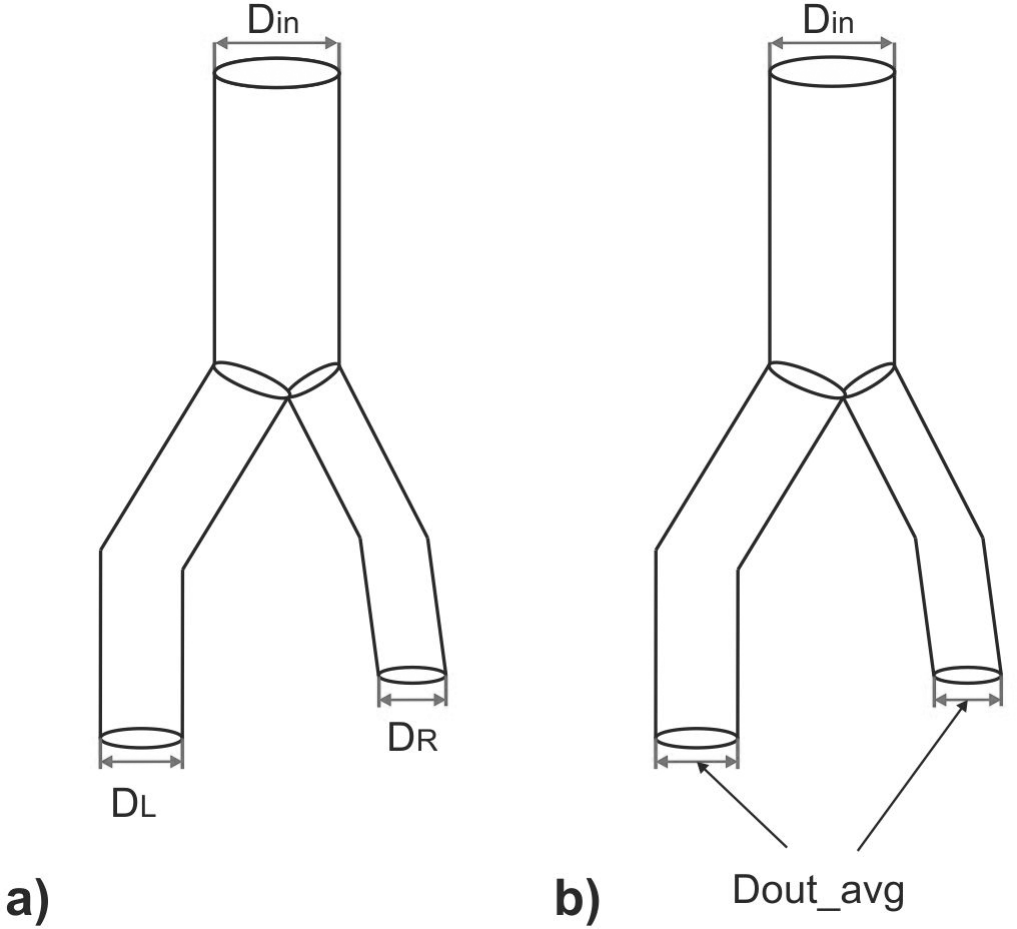
Schematic representation of the diameters analyzed in the stent-graft. *D*_*L*_−outlet diameter of the left branch of the stent-graft, *D*_*R*_−outlet diameter of the right branch of the stent-graft, *D*_*in*_−inlet diameter of the stent-graft, *D*_*out_avg*_−arithmetic average of both outlet diameters in the iliac part of the stent-graft.

Fig 2A and 2B show schematically the analyzed diameters of the stent-graft (Fig 2A), and a relation of the inlet diameter and average values of both outlet diameters in the stent-graft (Fig 2B).

**Fig 3:**
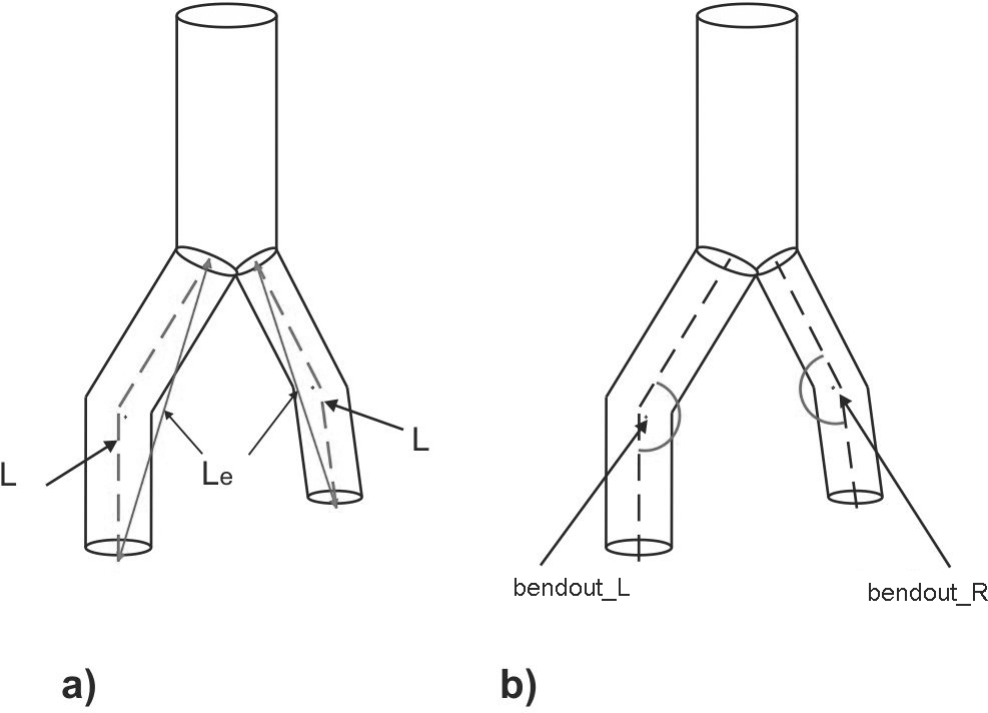
Schematic representation of the analyzed geometric parameters: a) tortuosity, b) bends in the stent-graft branches. *L*–length of the branch calculated in its axis, *L*_*e*_*−*distance between two parallel planes (inlet/outlet in the stent-graft branch), *bend*_*out_L*_−bend in the left branch of the stent-graft, *bend*_*out_R*_−bend in the right branch of the stent-graft.

Fig 3A and 3B show schematically the analyzed parameters including tortuosity and bends in the iliac part of the analyzed stent-grafts. We defined tortuosity as spiral twisting of the flow around the flow direction. Bends were described as the largest angulation in distal part of a stent-graft.

Geometric parameters used in the calculations were selected basing on the criteria used by the surgeons before implantation of a stent-graft. These criteria included the diameter of aneurysm neck and the diameters of iliac arteries, i.e. places in which the upper and lower part of the stent-graft was mounted, bends in the iliac arteries, and the ratio of the length of the common part of the stent-graft compared to the length of branches was studied.

A relation between deformations of branches in stent-graft and thrombus risk was analyzed for tortuosities and bends. Tortuosity of the stent-graft branches was determined as a relation of the distance *(L*_*e*_*)* between two parallel planes (inlet/outlet in the endoprosthesis branch) to the length of branch *(L)* calculated in its axis. A ratio of the left branch tortuosity (*T*_*L*_*)* to the right branch (*T*_*R*_*)* for which the fewest cases of endoprostheses for which the thrombus occurred was classified as optimal.

The second analyzed parameter which determined the deformation of the stent-graft branches was bends. We measured bends assuming that the straight branch corresponded to the angle 180°, while a completely bent branch corresponded to the angle 0°. In each endoprosthesis branch we selected the biggest bend from all bends occurring in the branch and used it for further simulations. Finally, we determined the relation of bends in both stent-graft branches (*bend*_*out_L*_*/bend*_*out_R*_*)* and we searched for a range of bending at which thrombus occurrence was less frequent.

### Statistical analysis

Statistical analysis was performed using GraphPad Prism Version 5.01 (GraphPad Software; San Diego, California, USA). Values were presented as mean±SEM. Correlations were evaluated with the Spearman rank correlation test or Pearson test. Data were considered as significantly different when p<0.05 unless otherwise noted.

## Results

Analysis of CFD results indicated that tthrombus was observed in 68% of stent-grafts when *Hct* = 40% (similar *Hct* value as in patients), while at *Hct* = 50% thrombus occurred in 83% of stent-grafts. Comparison of CFD results and medical data indicated that thrombus was reconstructed each time in the patient with thrombus originally diagnosed for *Hct* = 40%. Increasing of *Hct* value increased the number of patients with thrombus inside endovascular prosthesis.

### Relation of volumes

**Fig 4:**
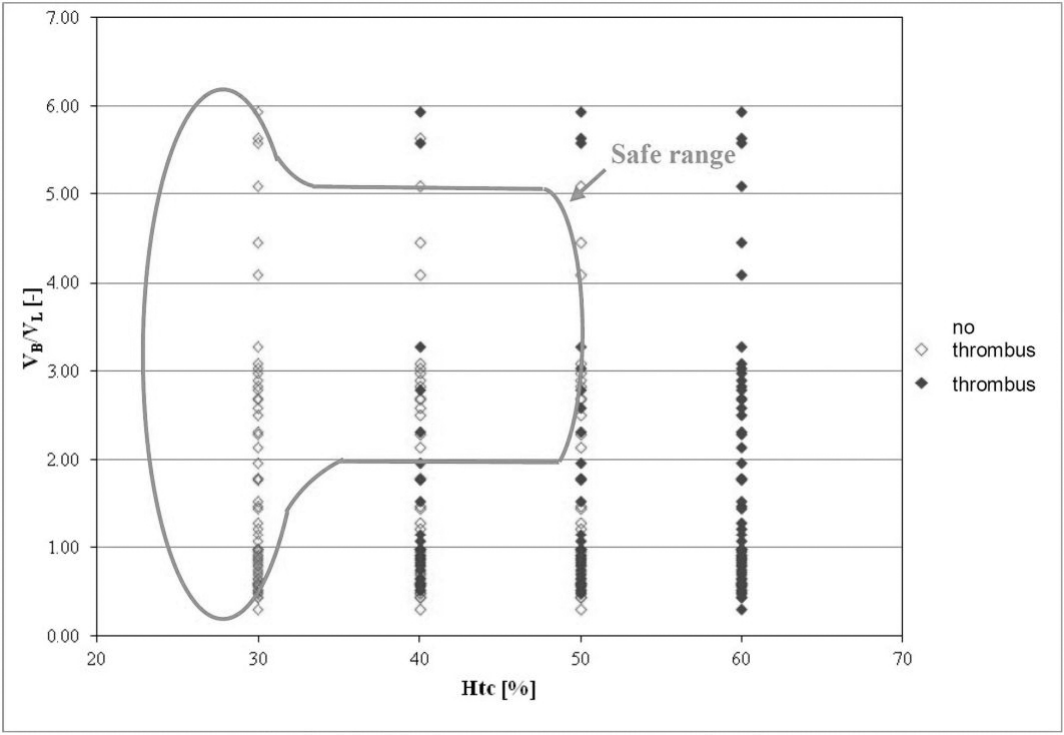
The relation of the volume of upper part of the stent-graft *(V*_*B*_*)* to the volume of branches in stent-graft *(V*_*L*_*)* presented as a function of *Hct*. Stent-grafts in which thrombus did not occur are marked in red; stent-grafts with thrombus are marked in blue; green color denotes the area in which the fewest clotted stent-grafts were observed.

First, the relation of aortic implant volume and thrombus risk was analyzed. We observed that with increasing *Hct* the number of endoprostheses with thrombus was rising (Fig 4).

The lowest risk of thrombus formation was observed when the volume of the upper part of the stent-graft (*V*_*B*_) was 2 to 5 times bigger than the volume of branches (*V*_*L*_) (Fig 4). For this range thrombus was observed in 9.3% of cases when *Hct* = 40%, and in 14% of cases when *Hct* = 50% (Fig 4). Verification of received results indicated that for *Hct* = 40% and the same range of analyzed geometric parameter approximately 80% of CFD results were in line with clinical cases.

### Relation of diameters

Next analyzed parameter was branch asymmetry (inlet diameter (*D*_*in*_) and outlet diameters (*D*_*L*_) and (*D*_*R*_)). Results showed that thrombus occurred more frequently in the geometries in which the diameters of both branches highly differed from each other. The risk of thrombus was lowest when diameters of both branches ranged from 0.8 to 1. Then, thrombus occurred in 16.7% of the stent-grafts at *Hct* = 40% and in 23% of the stent-grafts at *Hct* = 50%. Verification of received results indicated that for *Hct* = 40% and the same range of analyzed geometric parameter approximately 80% of CFD results were in line with clinical cases.

Our analysis of the relation of the upper to lower part of an endoprosthesis (Fig 4) showed that the process of thrombus formation was determined by spatial configuration of iliac and upper parts of the endoprosthesis. Therefore, in the next step, we analyzed the relation of inlet diameter (*D*_*inlet*_) to the arithmetic mean of both outlet diameters (*D*_*out_avg*_). Like in the case of the parameters analyzed previously, an increase in blood viscosity caused an increase of the number of stent-grafts with thrombus. The smallest risk of thrombus formation occurred when the analyzed parameter was in the range from 1.2 to 2. Then, thrombus was reported in 9,3% of the stent-grafts at *Hct* = 40% and in 17% of the endoprostheses at *Hct* = 50%. In conclusion, the risk of thrombus formation was the smallest when the diameter of the upper part of the endoprosthesis was 1.2 to 2 times of the diameters of its outlets. Verification of received results indicated that for *Hct* = 40% and the same range of analyzed geometric parameter approximately 80% of CFD results were in line with clinical cases.

### Relation of deformations

**Fig 5:**
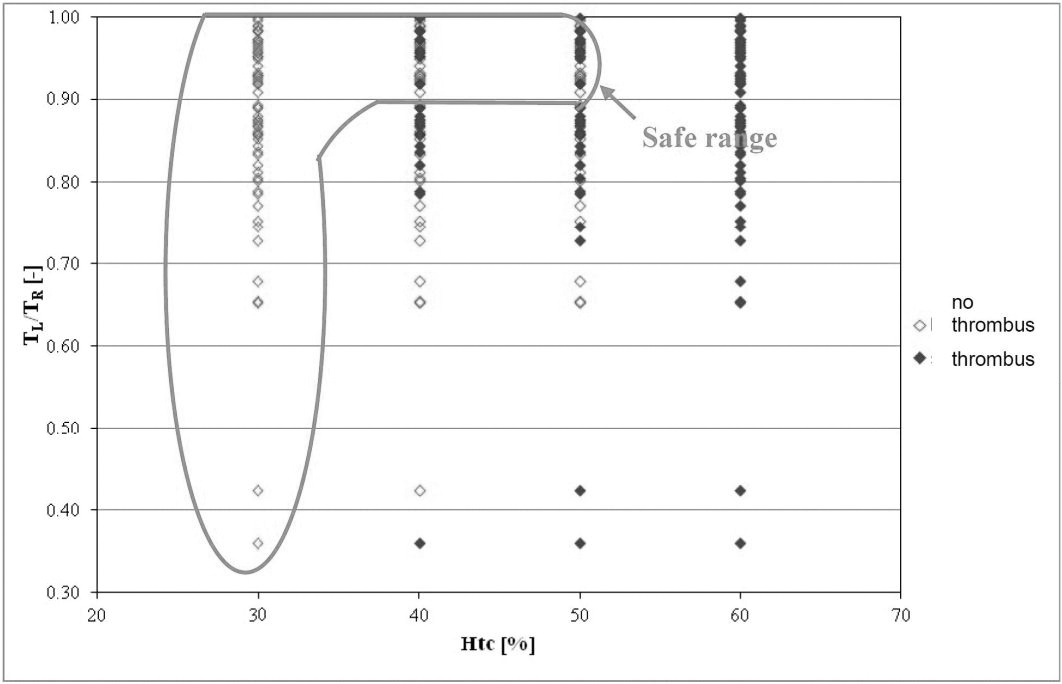
The relation of tortuosity (*T*_*L*_*/T*_*R*_) in the stent-graft branches as a function of *Hct*. Stent-grafts in which thrombus did not occur are marked in red; stent-grafts with thrombus are marked in blue; green color denotes the area in which the fewest clotted stent-grafts were observed.

**Fig 6:**
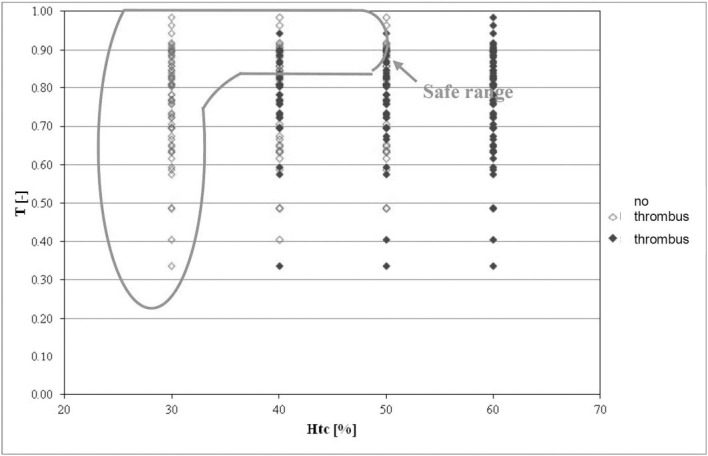
The relation of tortuosity *T* measured in the stent-graft branches as a function of *Hct*. Stent-grafts in which thrombus did not occur are marked in red; stent-grafts with thrombus are marked in blue; green color denotes the area in which the fewest clotted stent-grafts were observed.

The last of the analyzed parameters were deformations of the stent-graft, i.e. bends and tortuosity in the iliac part of the endoprosthesis. Tortuosity of the stent-graft branches were analyzed as a relation of tortuosity of both endoprosthesis branches in a function of *Hct* (Fig 5). Hence, the region of tortuosity for which thrombus was less frequently observed was in the range of 0.9 to 1. For this range, thrombus occurred in 26% and 37% of cases when *Hct* was 40%, 50%, respectively. Having that information we attempted to determine an optimum range of tortuosities in the iliac part of a stent-graft. Fig 6 shows that the area in which the lowest number of endoprostheses where thrombus occurred was in the range from 0.85 to 1, and then thrombus appeared in 17% of stent-grafts at *Hct* = 40% and in 22% of the stent-grafts at *Hct* = 50%. Verification of received results indicated that for *Hct* = 40% and the same range of analyzed geometric parameter approximately 91% of CFD results were in line with clinical cases measured with AngioCT.

**Fig 7:**
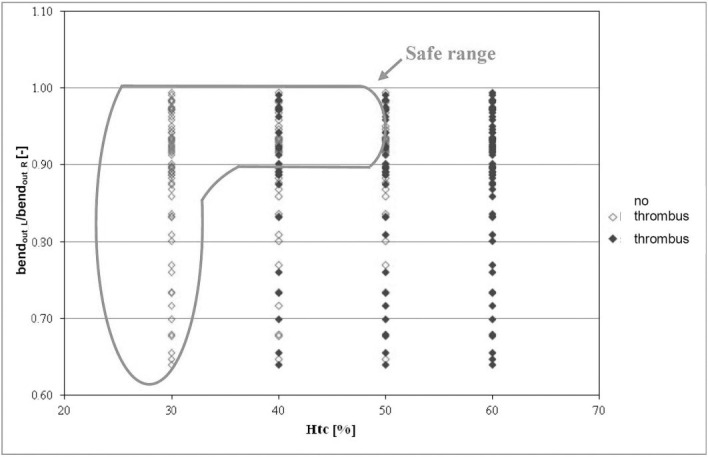
The relation of bends (*bend*_*out_L*_*/bend*_*out_R*_) in the stent-graft branches as a function of *Hct*. Stent-grafts in which thrombus did not occur are marked in red; stent-grafts with thrombus are marked in blue; green color denotes the area in which the fewest clotted stent-grafts were observed.

Finally, we analyzed the relation between thrombus formation and the appearance of bends in both branches of a stent-graft (*bend*_*out_L*_*/bend*_*out_R*_) (Fig 7).

**Fig 8:**
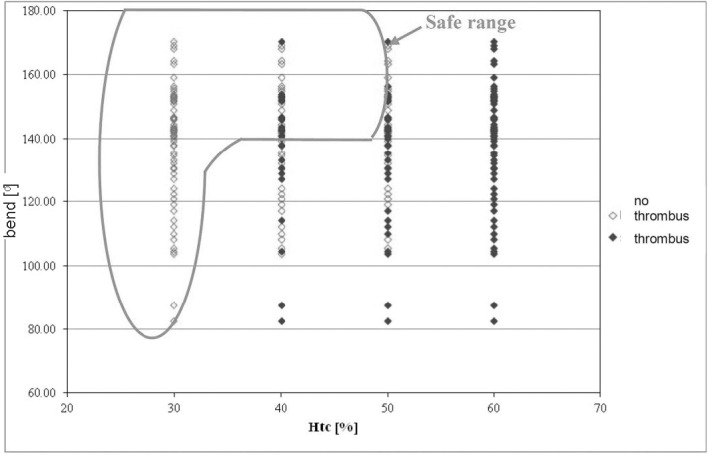
The relation of bends measured in the stent-graft branches as a function of *Hct*. Stent-grafts in which thrombus did not occur are marked in red; stent-grafts with thrombus are marked in blue; green color denotes the area in which the fewest clotted stent-grafts were observed.

The area, in which the fewest endoprostheses with thrombus were reported, ranged from 0.9 to 1. Then, at *Hct* = 40% thrombus was observed in 22% cases and at *Hct* = 50% in 31% of cases. Verification of received results indicated that for *Hct* = 40% and the same range of analyzed geometric parameter approximately 91% of CFD results were in line with clinical cases measured with AngioCT. Similarly as for tortuosity, we searched for an optimum range of bending with the lowest risk of thrombus formation. We indicated that the lowest risk of thrombus formation is for bends ranging from 140° to 180° (Fig 8).

The geometric parameters of stent-grafts which determined the optimum spatial configuration of the stent-graft were gathered in Table 2.

**Table 2:** Optimum geometric parameters of the stent-graft. V_B_—the volume of upper part of the stent-graft, V_L_—the volume of lower part of the stent-graft, D_L_−the left outlet diameters of iliac part of the stent-graft, D_R_—the right outlet diameters of iliac part of the stent-graft, D_in_—inlet diameter, D_out_avg_—average diameter at the outlet from the stent-graft, T–Tortuosity, T_L_—the tortuosity of left branch in the stent-graft, T_R_−the tortuosity of left branch in the stent-graft, Bend–Bend in the iliac part of the stent-graft, bend_out_L_−the bend in left branch of the stent-graft, bend_out_R_−the bend in left branch of the stent-graft.

Parameter	Range
**V**_**B**_**/V**_**L**_	2 ÷ 5
**D**_**L**_**/D**_**R**_	0.8 ÷ 1
**D**_**in**_**/D**_**out_avg**_	1.2 ÷ 2
**T**_**L**_**/T**_**R**_	0.9 ÷ 1
**T**	0.85 ÷ 1
**bend**_**out_L**_**/bend**_**out_R**_	0.9 ÷ 1
**Bend**	140° ÷ 180°

## Discussion

Our results showed that among the analyzed parameters the most important for thrombus appearance were tortuosity and angular bends of a stent-graft. Even with small values of tortuosity and angular bends in the iliac part of the prosthesis thrombus was restored in 22% to 31% of cases indicating these parameters as dominant for thrombus appearance.

Our previous reports showed that implantation of the unjustly fitted prosthesis correlates with the high risk of thrombus formation [2, 22]. This is in line with other authors who emphasized that inaccurate fitting of the aortic implant can lead to blood stagnation contributing to the formation of thrombus [13, 23]. The deposition of solid components of blood on the blood vessel wall is a natural process that appears mainly in the places of stent-graft bifurcation, as the bifurcation alters blood hemodynamic and increases the risk of thrombus formation [24]. Moreover, our results indicated that main part of a stent-graft should be larger compare to the iliac part to decrease the risk of thrombus development. Similarly, observation had Chong et al. [3] and Wu et al. [25] who indicated that when the volume of upper part of the endoprosthesis was bigger than the volume of lower part, there were changes in the blood flow velocity which increased the risk of thrombus formation. On the contrary Katritsis et al. [26], in the clinical studies, observed that the risk of thrombus formation increases when the main part of the endoprosthesis differed significantly from its branches, e.g. a big difference in cross-sectional areas. They indicated that the difference is often due to the mismatch of prosthesis or the use of a too long common part. For instance the mismatch of prosthesis related to a too large cross-sectional area of the upper part in relation to the cross section of branches, may result in a decrease of blood flow velocity and increased the risk of thrombus formation. Moreover, when cross-sectional areas of the upper and lower part of the stent-graft are comparable blood flow is uniform [26].

The next parameters which may disturb blood flow hemodynamic are angular bends and tortuosity of the iliac part of the endovascular prosthesis. We indicated in our study that tortuosity higher than 60% and angular bends higher than 60° increase the risk of thrombus formation. It is in line with Demanget et al. [27] who noted that the angle of the stent-graft bend smaller than 90° had no significant effect on the change in the implant cross-sectional area. The authors analyzed two types of stent-grafts differing in the construction of the metal scaffold which affected elasticity of the prosthesis. They presented that if the angular bend is ≤ 90° the prosthesis diameter does not change. When the angular bends exceed 90° cross-sectional area of the prosthesis is significantly reduced which may lead to the deposition of thrombocytes on the vessel wall, and in a consequence, to thrombus formation [27]. Similarly, Morris et al. [28] showed that the more complicated shape of the vessel the higher the risk of stent-graft displacement and bending of the prostheses. Moreover, Wegener et al. [29] showed that the formation of bends and tortuosity in the prostheses is determined by the inlet and outlet diameters and position of the stent-graft. Significant differences in the inlet and outlet diameters of the stent-graft cause that during blood flow on the prosthesis wall high values of wall shear stress are reported and may lead to displacement of the implant, and eventually to the deformation of its shape and formation of bends. It is also possible that in strongly bent prostheses one of force components will have a dominant effect and may cause displacement of the prosthesis and its bending [29].

A dynamic development of endovascular techniques used in abdominal aortic aneurysm treatment indicates constant improvement of stent-grafts' geometry [30, 31]. We believe that basing on AngioCT data and geometric properties of the aorta it will be possible to personalize the process of stent-graft application. Knowing aorta's geometric parameters surgeons could decide if particular stent-graft is appropriate for the patient. Furthermore, with the use of 3d reconstruction algorithms and CFD simulations it could be possible to create 3d model of blood hemodynamic including tortuous anatomy of particular patient and to check which stent-graft will have the lowest risk of postoperative complications. This could be a next step in patient-based medicine.

It was confirmed that the risk of occlusion is higher if the lumen of external iliac artery is below 10 mm and it has tortuous course [11]. On the other hand, no beneficial effects of statins on the outcome of treatment of thrombosis in aneurysm were proven [32]. There was also a negative effect of smoking and renal failure on the risk of closing of the prosthesis [33, 34]. Moreover, due to particular management standards, some factors cannot be treated as variables e.g. all patients received standard antiplatelet therapy according to the guidelines. It is certain that administration of antiplatelet/anticoagulant therapy decrease the risk of complications, however in this case we cannot compare it with patients who did not received such a therapy.

The major limitation of this study is a finite number of analyzed risk factors of a thrombus formation. There are well defined risk factors (e.g. smoking) and another potential ones (e.g. native aorto-iliac anatomy configuration) that we did not addressed. However, our aim was to focus on factors associated with intervention procedure. Consequently, in further research on bigger groups of patients, risk factors that are well known and also this newly presented by our team can be included into analysis.

## Conclusions

Our study indicated that to minimize the risk of thrombus formation in the stent-graft, the volume of upper part of the endoprosthesis should be two to five times bigger than the volume of the iliac part. Inlet diameter of the stent-graft should be 1.2 to 2 times bigger than the diameters of branches of the endoprosthesis.

Among the analyzed parameters, the most influential on thrombus formation are tortuosities and bends of the stent-graft. Even for small tortuosities and bends of the prosthesis thrombus was observed, in 22 to 31% cases. Hence, these seem to be the dominant parameters determining blood clotting which should be under special attention during stent-graft recommendation and patients’ follow-ups.
